# Spectral Domain – Optical Coherence Tomography findings in Triple-A Syndrome – A case series from Pakistan

**DOI:** 10.12669/pjms.37.1.3310

**Published:** 2021

**Authors:** Javeria Nasir, Anum Javed, Owais Arshad, Mohammad Hanif Chatni

**Affiliations:** 1 Dr. Javeria Nasir, MBBS, MCPS. Department of Ophthalmology, Patel Hospital, Karachi, Pakistan; 2 Dr. Owais Arshad, MBBS, FCPS, MRCS, FICO. Department of Ophthalmology, Patel Hospital, Karachi, Pakistan; 3 Dr. Anum Javed, MBBS. Department of Ophthalmology, Patel Hospital, Karachi, Pakistan; 4 Dr. Mohammad Hanif Chatni, MBBS, FRCS. Department of Ophthalmology, Patel Hospital, Karachi, Pakistan

**Keywords:** Triple A Syndrome, Optic atrophy, SD-OCT

## Abstract

Triple A Syndrome is an autosomal recessive entity involving multiple systems usually characterized by adrenal insufficiency, alacrimia and achalasia. The disease features include variable degrees of neurological and neuro-ophthalmic manifestations. Protein ALADIN encoded by the AAAS gene is found to be defective in Triple A Syndrome. Here we discuss a case series of five patients diagnosed as Triple A Syndrome. Clinically there was variable degree of optic atrophy in all the cases, which was further confirmed with spectral domain Optical Coherence Tomography The aim of this study was to publish the OCT based ONFL graphs of these unique cases, so that being an ophthalmologist we can take a multidisciplinary approach and decisions accordingly.

## INTRODUCTION

Triple A Syndrome (TAS) is a multi-systemic disease with typical features of ACTH-resistance causing adrenal insufficiency, alacrimia and esophageal atresia.^1^ These classic findings can be seen in around two third of the patients with one third patients also showing abnormalities of autonomic nervous system, due to which some authors might use the term “4A syndrome”.^2^ Common neurological findings include dysautonomia, cognitive decline, pyramidal and neuro-ophthalmic signs. The syndrome is inherited in an autosomal recessive pattern, due to mutations in the AAAS gene coding nucleoporin ALADIN.^3^

Ophthalmic involvement may either be due to autonomic dysfunction of the lacrimal apparatus or structural compromise of the ocular surface. Alacrimia, lacrimal gland atrophy and keratoconjunctivitis sicca are known manifestations in the anterior segment.^4^ Furthermore, optic atrophy, defective pupillary reflexes, anisocoria and Horner’s syndrome are additional findings.^5^

However due to decreased prevalence of the disease along with extreme phenotypic heterogeneity, early diagnosis of the disease is challenging for the primary physician OCT is a two-dimensional cross-sectional non-invasive imaging technique. It has revolutionized ocular imaging ever since Huang *et al*. described it in 1991.^6^ Spectral domain OCT provides a greater number of images per unit area with better resolution power.^1^ SD- OCT findings in TAS have not been studied previously in detail. The present case series highlights the OCT based ONFL (optic nerve fibre layer) patterns found in TAS, all of which were diagnosed on the basis of ACTH stimulation test and baseline levels of serum ACTH & cortisol together, along with the classical clinical trial of achalasia, alacrimia and adrenal insufficiency. Although there have been few case reports on TAS from our country, 1,7 to the best of our knowledge, this is the first case series being reported from Pakistan, regarding these OCT findings.

### Case-1

**Fig.1a & 1b:**
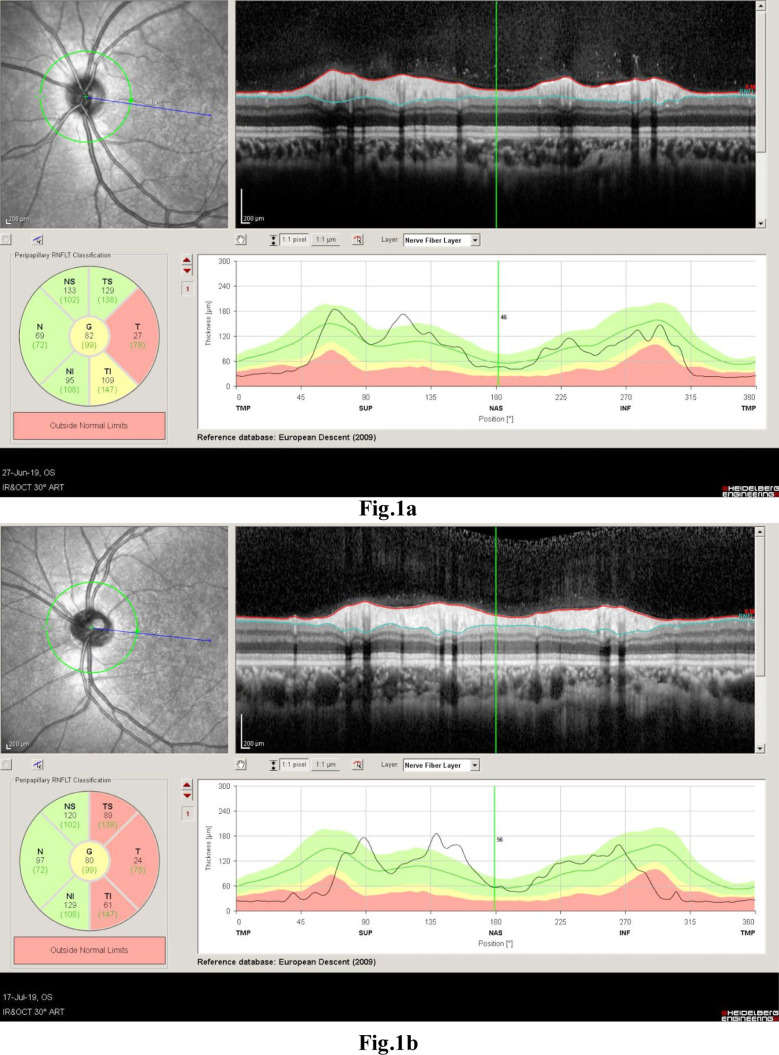
Graphical Presenation of ONFL on SD-OCT, corresponding to the temporal optic atrophy.

A four-year-old female child suspected as TAS presented to us for the evaluation of alacrimia. Her parents noticed that the she was unable to produce tears since the early days of her life. Ophthalmic examination was unremarkable, apart from alacrimia (confirmed on Schirmer test showed less than 5mm of wetting of filter paper after 5 minutes) and temporal optic atrophy (on SD-OCT, RNFL showed thinning of layers in temporal region of both eyes) (Fig.1).

### Case-2

Eight-year-old girl, suspected of TAS due to increased serum cortisol levels, having complains of dysphagia, blurry vision and itching in both eyes for the past two years was referred to the eye OPD for assessment of ocular symptoms. Except for the decreased tear meniscus levels (Schirmer test showed less than 5mm of wetting of filter paper after five minutes) and mild superficial punctate keratitis superiorly in both corneas, anterior segment examination was unremarkable. Both eyes revealed optic disc pallor temporally which were further confirmed on SD-OCT.

### Case-3

A seven-year-old girl came to eye clinic for examination regarding ocular findings of TAS. She had mild blurry vision for the past six months. There was loss of corneal luster indicating corneal dryness in both eyes. Schirmer test showed 3mm of wetting of filter paper after five minutes. Fundoscopy revealed bilateral optic disc nasal and temporal rim pallor. SD-OCT also showed reduced thickness of RNFL layers in the papilla-macular bundle.

### Case-4

**Fig.2a & 2b:**
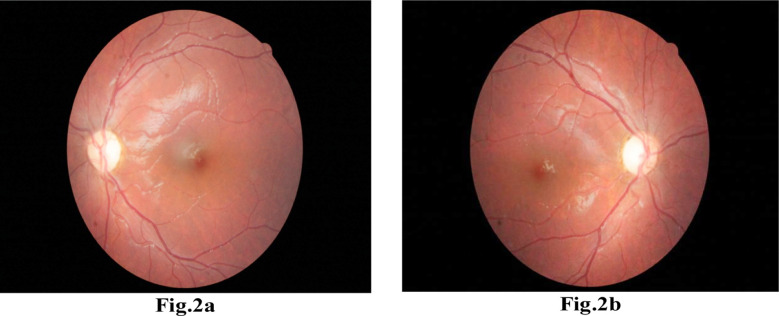
Depicting the typical temporal optic disc atrophy.

A nine year old boy, suspected case of TAS was referred to us for the evaluation of ocular findings in TAS, similar to Case 1. Ophthalmic examination was normal in both eyes. However, optic discs appeared suspicious in both eyes with a pale looking neuro-retinal rim. SD-OCT showed decreased RNFL thickness at the superior pole of both eyes. (Fig.2)

### Case-5

A four-year-old girl presented for work-up of alacrimia. She was the younger sister of Case-1. Slit lamp examination was performed. Schirmer test showed zero wetting of filter paper indicating absolute alacrimia. Indirect fundoscopy revealed bilateral temporal optic atrophy. SD-OCT could not be performed due to poor cooperation.

Other than dry eyes, there was no refractive error and the pupillary responses were normal for all the children. We were able to perform optic nerve functions in three of our patients, which included visual acuity, color vision, relative afferent pupillary defect (RAPD) and confrontation test. All five cases clinically showed temporal optic atrophy on slit lamp examination, indirect ophthalmoscopy and OCT.

## DISCUSSION

TAS was first described in 1978, although its genetic and clinical characteristics have been studied widely but current literature is limited to case reports and case series mainly. The foremost clinical features of TAS include alacrimia, ACTH resistance, adrenal inadequacy and achalasia.^8^ Alacrimia is usually the first and most consistent finding at presentation as noted in our case series. Four out of five patients presented with dry eye symptoms. Achalasia and ACTH resistance may manifest over a variable span of time.Other ophthalmic findings may include corneal melting, keratoconjuctivitis sicca, lacrimal gland atrophy, pupillary abnormalities (tonic pupils, sluggish pupils), lazy eye, accommodative dysregulation and optic atrophy.^9^’^10^ Keeping in mind the association of “dry eyes” in children with achalasia or adrenal insufficiency,^11^ all five children were referred to us for ophthalmic evaluation. Birth history (antenatal, natal & post natal), past ocular history, family history, nutritional history and socioeconomic history all were insignificant. Clinically pupils were normally reactive and no relative afferent pupillary defect (RAPD) noted in all patients. Three out of five children were cooperative in terms of optic nerve functions. Apart from decreased lacrimation and typical characteristic optic atrophy, the rest of the eye examination was normal in all cases. No infectious, inflammatory, nutritional and environmental causes were found in these patients as discussed by Chinta et al.^12^

The most interesting observation in our case series was the presence of mild to moderate degree of bilateral, characteristic pattern of optic atrophy in all cases invariably. The clinical findings of fundoscopy were confirmed with SD- OCT. We performed OCT’s in four out of five patients. RNFL thinning was present in OCT scans exactly corresponding to our clinical findings of disc pallor as seen in specific quadrant of optic nerve head on fundoscopy. Authors believe that these bilateral, symmetrical, temporal optic atrophy patterns are characteristics of this syndrome. It is difficult to say whether this atrophy is a physiological or pathological variant of optic disc. It needs a larger number of patients and a prospective follow-up on the ONFL pattern on OCT. Moreover, ONFL pattern in children follow the same pattern in adults i.e. thick at the poles and thinner in the nasal and temporal quadrants.^13^.^14^ Authors want to publish this characteristic ONFL pattern of optic atrophy internationally from this part of world. In view of this; we believe that it could be the diagnostic clue of these types of the syndromes. This *typical pattern* of optic atrophy seem to be associated with TAS. The authors feel that after following the OCT findings of our cases, this feature can be added as a diagnostic pointer in cases of TAS. At one year follow-up, OCT was repeated which showed the same RNFL values as before, signifying the non-progressive nature of TAS-associated optic atrophy.

### Limitations of the study

We were unable to perform the standard automated perimetry (SAP) test in four of our patients due to age limitation. Another limitation of our study was the inability to check all the optic nerve functions in two of our patients, due to the same reasons mentioned above.

## CONCLUSION

With this case series authors conclude that patients with tripleA syndrome might present with varied ocular features including alacrimia, anisocoria and optic atrophy. Ophthalmologist must not only be vigilant about the systemic associations when receiving children with unexplained dry eyes or alacrimia, but also be proactive while evaluating diagnosed cases of TAS as early detection of associated optic atrophy and its progression may affect the long-term visual prognosis of an individual. Moreover, visual field testing is an important diagnostic modality that can help us monitor visual function at subsequent follow-up visits of these patients with progressively declining ONFL, if that was to occur.

### Author’s Contribution:

**JN:** Conceived, designed and did statistical analysis & editing of manuscript.

**AJ, MOA, JN & MHC:** Did data collection and manuscript writing.

**MHC:** Did review and final approval of manuscript.

**MOA & MHC:** Responsible and accountable for the accuracy/integrity of the work.
